# Job satisfaction and depression in the Spanish Society of Periodontology and Research (SEPA) members, and their relation to the burnout syndrome. Creation of a structural model

**DOI:** 10.4317/medoral.17478

**Published:** 2012-05-01

**Authors:** Mercedes Reyes-Torres, José V. Ríos-Santos, Ana López-Jiménez, Mariano Herrero-Climent, Pedro Bullón

**Affiliations:** 1 Department of Stomatology; 2 Department of Experimental Psychology University of Seville (Spain)

## Abstract

Objective: This study is aimed at getting to know the existing relationship between the dimensions of the burnout syndrome and job satisfaction, on one hand, and depressive feelings on the other through the creation of a structural model aimed at relating all these concepts on a sample of Spanish periodontists. Study design: The initial sample comprised 284 individuals, who represented 20% of the members of the Spanish Society of Periodontology and Research (www.SEPA.es). These individuals were chosen randomly by means of stratified sampling with proportional affixation by their autonomous community of residence. All participants were sent by post the MBI, CET and job-satisfaction questionnaires. The software package used for data analysis was LISREL v. 8.7 by checking models of structural equations so as to prove the proposed model’s adjustment. Results: The total number of answered questionnaires was 170 (59.85%). A positive relation was observed between emotional tiredness and depersonalization and depression. However, this dimension correlated negatively with job satisfaction and self-realization. Conclusions: The obtained results show that, in this sample of periodontists, job satisfaction acts as a modulator in the transition from emotional tiredness to depression.

** Key words:**Burnout, depression, periodontology.

## Introduction

Stress in the working world is associated to increased numbers of working accidents and reduced productivity. Continuous exposure to stress may produce a wide range of harmful consequences for the workers’ health, thus provoking physical and mental disorders that lead to conditions such as the burnout syndrome. Its symptoms are characterized by three clearly-differentiated dimensions: emotional tiredness, depersonalization, and reduced self-realization, which lead to remarkably reduced working performance and productivity. In spite of being different gnoseologic entities, the burnout syndrome, in the long run, may provoke the appearance of the depressive syndrome, if the conditions that lead to its appearance hold throughout time, thus soaking the individual’s all other life aspects with all these harmful symptoms.

Most of the consequences of working stress in organizations can be associated to the burnout syndrome. Indeed, reduced job satisfaction is among those with the greatest impact on working objectives and performance.

Job satisfaction can be easily defined as people’s more or less defined idea on how they believe that their work —together with its most important aspects— should develop. Comparing these ideas with their reality renders an assessment and a resulting attitude: greater or lower satisfaction levels.

Emotional tiredness and depersonalization are negatively associated to job satisfaction, while self-realization is positively associated to it. However, studies do not precise whether job satisfaction is an antecedent or a consequence of the burnout syndrome.

All these conditions have been studied in different health populations (1,2) though references on dentists are rather scarce (3,4). The only research focused on the burnout syndrome in periodontists in Spain was recently carried out by our own research group (5).

One of the objectives of this study was the completion of a structural model to interrelate the three dimensions of the burnout syndrome (that is, emotional tiredness, depersonalization, and self-realization to depression and low job satisfaction levels among periodontists).

## Material and Methods

-Sample

To achieve the main aim of this research, the members of the Spanish Society of Periodontology and Research (SEPA) were taken as the sample population.

**Figure 1:**
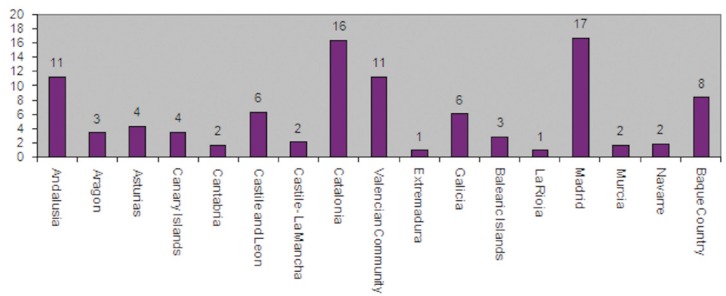
Distribution of SEPA members by percentages and autonomous communities.

The number of members of SEPA, as of November 2nd, 2004, was 1419. Since we are dealing with a reduced population, the sample was decided to reach 20% of that population to reduce estimation errors —thus, an initial sample of 284 individuals was registered. These individuals were chosen by stratified random sampling with proportional affixation by autonomous community (Fig. 1).

-Instruments 

We used a series of questionnaires, which were posted to the participating SEPA members. They were sent a document containing an introduction page that explained the nature and objectives of the study and asked them for collaboration, a questionnaire of sociodemographic and working variables, and the Maslach & Jackson Burnout Inventory (MBI (6), 1986), and the Brief Four-dimensional Structural Questionnaire for Depression (Brief CET-DE). The impact of stress on job satisfaction was assessed by means of a specific questionnaire contributed by Meliá (7).

-Procedure

All participants were posted an envelope by the University of Seville that contained an introduction letter by the research group, the questionnaire, and the instructions to fill it in. It also contained a stamped envelope to return the questionnaires. The participants were asked to post these documents to a collaborating notary, who collected all documents and remained anonymous so as to guarantee confidentiality in the returned questionnaires, which is an essential requirement for the study. A total number of 284 questionnaires were sent. The notary gave the research group the list of those who had finally returned the questionnaire so as to contact them on the phone.

-Statistical analysis

The software package used for statistical analysis was LISREL v. 8.7, which was used to test models of structural equations to assess the proposed model’s adjustment. When data of the observed variables are collected for the theoretical model, LISREL can then be used for assessment purposes.

## Results

The total number of returned questionnaires was 170 (59.85%).

Using the model’s construction strategy, the proposed model was estimated, eliminating insignificant direct effects as long as they did not deteriorate global adjustment measures. This model obtained different global values for different tests, which justifies its adjustment —chi2 was 0.41, RMSA was 0.02, GFI was 0.99, and AGFI reached 0.96.

**Figure 2:**
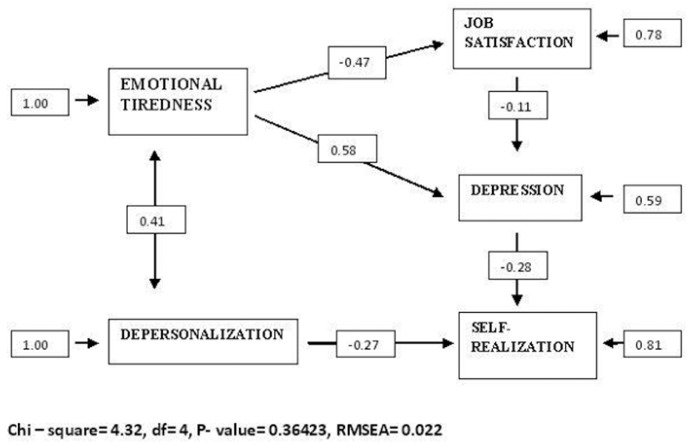
Structural model.

All the relations observed between the structural model’s variables, shown in figure 2, were significant for p < 0.05, except the relation between depression and self-realization, with marginal signification.

A positive relation was observed between emotional tiredness and depersonalization (chi2 = 0.41) and depression (chi2 = 0.48). However, this dimension is negatively related to job satisfaction (chi2 = -0.47).

The results obtained indicate that job satisfaction acts as a mediator between emotional tiredness and depression. As observed in figure 2, depression and self-realization correlate negatively (chi2 = -0.28), as well as the latter variable and depersonalization (chi2 = -0.27).

## Discussion

This is the first study completed among Spanish periodontists aimed at relating, within a structural model, the dimensions of the burnout syndrome with depression and job satisfaction.

Research in dentistry is usually mainly focused on the patient’s health, thus neglecting the importance of health workers’ (dentists’) both mental and physical strength (8).

Therefore, the main aim of our research is getting to know the existing relation between the different conditions that affect the participants in the this study (i.e., SEPA members).

Analysis of the obtained results shows a positive relation between emotional tiredness and depression, as well as between the former and depersonalization.

Of course, the first dimension of the burnout syndrome can include similar symptoms to those observed in the depressive syndrome in the affected periodontists such as sadness or dejection, low self-esteem, appetite loss, weight or sleep-pattern changes, difficulties to concentrate, apathy, and feeling of guilt —thus resulting in damaged social and interpersonal functions (9). These results coincide with most studies completed on this professional field (see, for instance, Haakanen) (10).

Likewise, the self-realization dimension is completely opposite to the afore-defined concepts. However, the most interesting result among those put forward in this paper is related to the mediating role of job satisfaction between emotional tiredness and depression. That is, job satisfaction can play a modulating role in the transition from emotional tiredness to the depressive syndrome. Thus, obviously, a negative relation is observed between satisfaction and depression.

Graham (11) also completed a model based on a research on physicians in which the dimensions of the burnout syndrome were related to working stress, satisfaction and the sample’s psychiatric morbidity. It included the protective factor of job satisfaction, which was positively related to self-realization.

If the dentist profession is analyzed in detail, it can be observed that many factors can trigger working stress and thus favor the professional burnout syndrome. Some are not dentistry-specific, as they can be considered to be shared by some other occupations, such as economic-administrative pressures, business management requirements, planning payments to third parties, or dealing with the growing competition (12). However, there are some highly dentistry-specific factors such as periodontic procedures (13), working with anxious or distressed patients (14), visibility and patients’ limited access or negative, distrustful attitudes (which are becoming more and more common each day), and the fact of provoking pain. It must be taken into account that the patient that makes himself comfortable on the dental chair is usually seized with anxiety, which is also commonly transferred to the periodontist throughout the operation. Finally, not only clinical capacity is necessary, but the dentist also needs to get involved in the world of “marketing and productivity”, for which he receives no training along his academic life. Achieving this productivity entails covering a wider range of products and services within the same time unit, with the same operational costs and no quality reductions. All this means a greater amount of managerial and operational knowledge that, if employed incorrectly, may lead to economic failure and subsequent stress in periodontic professionals.

In spite of suffering emotional tiredness, provoked by the aforementioned stress triggers, it seems logical to think that some kind of factor, which modulates transition to depressive feelings, exists when the earliest burnout-syndrome symptoms appear. Our results point out that this factor is the feeling of satisfaction derived from professional activity. The existence of emotions in the periodontist that reflect wellbeing or satisfaction with the work completed on the patient, as well as the acquired knowledge that is put into practice, can modulate the subsequent appearance of a depressive syndrome.

These results are confirmed by comparison with those contributed by other studies, such as those completed by Ramírez (15) among surgeons, radiologists and digestive-health physicians in different British associations. They also coincide with Becker (16), who completed a study on gynecologists and obstetricians that showed a negative relation between job satisfaction and depression.

Our results contrast with the structural model achieved by Gil Monte (17), in which low job satisfaction appears as a mediator before job abandonment as well as a consequence of the burnout syndrome. According to this author, professional burnout is a mediating variable among the professional activity’s different stressing factors and consequences such as poor health. Job satisfaction does play a relevant role as a modulating key of the tendency to job abandonment.

To sum up, it must be pointed out that —although job satisfaction in dentistry seems to play a relevant role in the specialist’s transition from the burnout syndrome to depressive symptoms —further research shall determine how and when this evolution takes place so as to try to control these conditions and thus assure better health and working quality for periodontists.
